# Surgical repair of abdominal aortic aneurysms on the public health system in the largest city in Brazil: a descriptive analysis of in-hospital data on 2693 procedures over 10 years

**DOI:** 10.1590/1677-5449.202100872

**Published:** 2022-08-15

**Authors:** Marcelo Passos Teivelis, Marcelo Fiorelli Alexandrino da Silva, Nickolas Stabellini, Dafne Braga Diamante Leiderman, Claudia Szlejf, Edson Amaro, Nelson Wolosker

**Affiliations:** 1 Faculdade Israelita de Ciências da Saúde Albert Einstein – FICSAE, São Paulo, SP, Brasil.; 2 Universidade de São Paulo – USP, São Paulo, SP, Brasil.

**Keywords:** abdominal aortic aneurysm, public health, vascular diseases, aneurisma da aorta abdominal, saúde pública, doenças vasculares

## Abstract

**Background:**

From 1990 to 2015, mortality from aortic aneurysms increased 16.8% in Brazil. São Paulo is the largest city in Brazil and about 5 million people depend on the public health system there.

**Objectives:**

To conduct an epidemiological analysis of abdominal aortic aneurysm surgeries in the city of São Paulo.

**Methods:**

Infra-renal aortic aneurysm procedures performed over a decade (from 2008 to 2017) were studied using publicly-available platforms from the Unified Health System and DATASUS.

**Results:**

2693 procedures were analyzed; 66.73% were endovascular; 78.7% of patients were male; 70.7% were aged 65 years or more; 64.02% were elective hospital admissions. There were 288 in-hospital deaths (mortality: 10.69%). In-hospital mortality was lower for endovascular surgery than for open surgery; both for elective (4.13% versus 14.42%) and urgent (9.73% versus 27.94%) (p = 0.019) admissions. The highest volume hospital (n = 635) had the lowest in-hospital mortality (3.31%). USD 24,835,604.84 was paid; an average of $ 2,318.63 for elective open, $ 3,420.10 for emergency open, $ 12,157.35 for elective endovascular and $ 12,969.12 for urgent endovascular procedures. Endovascular procedure costs were statistically higher than the values paid for open surgeries (p <0.001).

**Conclusions:**

Endovascular surgeries were performed twice as often as open surgeries; they had shorter hospital stays and lower mortality.

## Resumo

**Contexto:**

No Brasil, a mortalidade por aneurisma de aorta aumentou 16,8% de 1990 a 2015. São Paulo é a maior cidade do Brasil, e cerca de 5 milhões de pessoas dependem do sistema público de saúde.

**Objetivos:**

Análise epidemiológica das cirurgias do aneurisma de aorta abdominal na cidade de São Paulo.

**Métodos:**

As cirurgias para correção do aneurisma de aorta infrarrenal realizadas no período de uma década (de 2008 a 2017) foram estudadas utilizando-se plataformas publicamente disponíveis do Sistema Único de Saúde e do Departamento de Informática do Sistema Único de Saúde.

**Resultados:**

Foram analisados ​​2.693 procedimentos, entre os quais 66,73% eram endovasculares. Entre os pacientes, houve predominância do sexo masculino (78,7%) e daqueles com 65 anos ou mais (70,7%). Um total de 64,02% eram admissões hospitalares eletivas. Ocorreram 288 óbitos hospitalares (mortalidade: 10,69%). A mortalidade durante a internação foi menor para cirurgia endovascular do que para cirurgia aberta tanto no contexto eletivo (4,13% *versus* 14,42%) quanto urgente (9,73% *versus* 27,94%) (p = 0,019). O maior volume (n = 635) apresentou menor mortalidade intra-hospitalar (3,31%). Foi pago um total de $24.835.604,84, sendo uma média de $2.318,63 para cirurgia abertura eletiva, $3.420,10 para cirurgia abertura de emergência, $12.157,35 para cirurgia endovascular eletiva e $12.969,12 para cirurgia endovascular na urgência. Os custos dos procedimentos endovasculares foram estatisticamente superiores aos valores pagos para as cirurgias abertas (p < 0,001).

**Conclusões:**

Foram realizadas duas vezes mais cirurgias endovasculares do que abertas, as quais apresentaram menor tempo de internação e menor mortalidade.

## INTRODUCTION

The most common site of aneurysmal dilatations of the abdominal aorta is infrarenal, close to the aortic bifurcation.^1^ Mortality after aneurysm rupture is high. It is estimated that approximately 80% of patients with ruptured aneurysms will die and approximately one-third of patients die before reaching hospital.^2^ In large trials, elective treatment of abdominal aneurysms had mortality rates ranging from approximately 1% for endovascular surgery to approximately 3.5% for open surgery, illustrating the importance of patients undergoing surgery before rupture.^3^

Currently, the therapeutic options for infrarenal abdominal aortic aneurysm (IAAA) are open treatment and endovascular treatment (EVAR). The first has been performed for over 60 years and the second for over 25 years and both are now considered well-established treatments. Several studies (DREAM, OVER, EVAR-1) have shown lower mortality in the first 30 days for the endovascular technique, with conflicting results for the difference in long-term mortality in patients treated with the different techniques.^4^^-^7

In Brazil, from 1990 to 2015, cardiovascular mortality from aortic aneurysm (in all segments, as classified in the death certificate as underlying cause of death) increased 16.8%; in women, this increase was 64.6%, while for men, there was a decrease of 2.4%.^8^ In Brazil, 75% of the population is served through the Unified Health System (SUS), a universal, equitable, and comprehensive tax-funded government system that performs 75% of highly complex procedures.^8^ The remainder of the population (25%) uses the private supplementary health system, where individuals or employers themselves pay for the health system.^9^ These treatments are not registered on government databases, even if they are performed in the same hospitals.

São Paulo is the largest city in Brazil and is 2nd in the country’s human development index ranking (0.783).^10^ It is estimated that there were more than 12 million inhabitants in the São Paulo municipality in 2016^11^ and more than 5 million of its inhabitants depend solely on the SUS.^9^ As a developed city, fewer people depend on the SUS than is the case in the national data. The other 7 million inhabitants depend on the non-public health system. A study conducted at the beginning of this century in the city of São Paulo found that approximately 1.8 to 3% of people aged 50 or older had abdominal aortic aneurysms.^12^ This range increases to 4.3 to 8% in men over 60.^12^ Moreover, because it is one of the most important cities in the country, a large number of patients from other municipalities and even from other states undergo surgery in São Paulo when they have complex vascular diseases.

There are no studies in our country that have evaluated the in-hospital outcomes of IAAA surgical treatment in a single municipality over a long period of time, compiling several variables.

The purpose of this study is to evaluate the frequency of IAAA surgical procedures performed by the public health system in the largest city in Brazil from 2008 to 2017.

## MATERIALS AND METHODS

Public-domain data on vascular and endovascular surgery procedures for IAAA repair performed from 2008 to 2017 were extracted from the TabNet platform, maintained by the Department of Informatics of the Unified Health System (DATASUS),^13^ which provides open-access data on procedures performed on the Brazilian public health system. Data is de-identified by DATASUS. Informed consent was therefore waived by our Institutional Review Board (IRB), as de-identification at the governmental source precludes identification of patients and means consent is not applicable. The study was approved by the Ethics Committee (process number 3067-17). Currently, DATASUS is a major provider of software solutions for state and municipal health departments, assisting the Brazilian Ministry of Health. SIGTAP (Management System of the Table of Procedures, Medicines, Orthoses, Prostheses and Special Materials of the SUS) provides information on which institutions linked to SUS perform each procedure.

Only hospitals accredited for vascular surgery were studied, because only data on accredited hospitals is available for analysis. Being “accredited” is a prerequisite for receiving payments from the SUS for surgeries performed. The platform has 22 possible selections for rows, 16 for columns, and 8 for content, providing 2816 possible formatting combinations, separated into monthly periods.

The variables selected for this analysis were gender, age group, municipality of residence, number of surgeries performed (total and by establishment), in-hospital mortality, length of stay at the establishment (divided into groups: 0 days, 1 day, 2 days, 3 to 6 days, and 7 days or more), mean length of stay in the intensive care unit (ICU), and sums paid out by the SUS over the years.

In all, 3 different procedures to treat abdominal aortic aneurysms were analyzed. These are identified through the codes of the SUS System of Procedures and Medicines and the OPM (orthotics, prostheses, and special materials) Table Management System. The following procedures were investigated: infrarenal abdominal aortic aneurysmectomy (code: 04.06.02.004-3), endovascular aneurysm repair/abdominal and iliac aortic dissection with bifurcated stent graft (code: 04.06.04.016-8), and endovascular aneurysm repair/abdominal aortic dissection with straight/conical stent graft (code: 04.06.04.015-0). To avoid including treatment for congenital malformations of the great arteries, all procedures classified as Q25 by the International Classification of Diseases (ICD) were excluded. Only the first letter and the first two numbers of the ICD code are available in the database. It is not possible to conduct analyses using the third ICD number.

We divided the patients into two groups: the open group, consisting of patients who underwent open surgery, and the endovascular group, consisting of patients who underwent endovascular surgery. In addition to this division, patients were also separated by the hospital admission administrative regime reported: elective or urgent.

The information used was obtained from public-access sites using computer programs to access the content with web scraping codes. While the data used are public domain, collecting all data manually would be time consuming, although technically feasible. To facilitate and expedite data collection, we used automatic navigation codes with programming assistance. These codes were programmed in the Python language (v. 2.7.13, Beaverton, Oregon, USA) on the Windows 10 Single Language operating system. The stages of collecting data, selecting fields on the platform, and later adjusting the tables were performed using the Selenium WebDriver (v. 3.1.8, Selenium HQ, various contributors around the world) and pandas packages (v. 2.7.13, Lambda Foundry, Inc. and PyData Development Team, New York, USA). The web scraping code has a main structure with 14 search phases (Appendix A) adaptable to the different filters available on the platform. We used the Mozilla Firefox browser (v. 59.0.2, Mountain - California - USA) and WebDriver.GeckoDriver (v 0.18.0, Mozilla Corporation, Bournemouth, England).

After collection, data were organized and grouped in a spreadsheet using the Microsoft Office Excel 2016® program (v. 16.0.4456.1003, Redmond - Washington - USA). The table was formatted so that the disease groups were placed side by side, containing, for each group, the following subdivisions: ranking by number of procedures performed at each of the establishments (ranked in descending order), total number of patients operated, intraoperative and in-hospital mortality (absolute and percentage), length of stay (in clusters), average length of stay in the ICU, and sums paid out by the SUS. Supplementary Material contains a video showing the computer program analyzing the data from the website database, as an example of part of the information acquisition process.

The amounts paid in Reais (the official Brazilian currency) were converted into US dollars at the exchange rate from December 31, 2012 (which is the mid-point between the first and last data analyzed). The hospitals were numbered, in descending order, by the total number of procedures performed. Thus, in our study, we were able to use the publicly-available data more simply and rapidly, analyzing data on a larger number of procedures and years.

The following tests were used for the statistical analysis: the chi-square test was used to investigate whether there was a change over the years in the number of procedures performed using each technique (trend test) and the Mann-Whitney test was used to compare mortality, hospital stay, and the values paid by the SUS between the open and endovascular groups. The method used to analyze length of ICU stay included a generalized estimating equation with normal distribution and identity link function. The level of statistical significance was p = 0.05 for all tests.

## RESULTS

In total, 2693 procedures performed in the city of São Paulo from 2008 to 2017 were analyzed. Most of the patients treated were male (78.7%). Most of the procedures were performed in elderly individuals aged 65 years or older (70.7% of the total). Almost two-thirds (62.7%) of the individuals operated had a registered residential address in the city of São Paulo.

Table 1 shows the number of procedures and percentage values per year according to the type of procedure (open or endovascular) and whether admission to hospital was elective or urgent.

**Table 1:** Absolute and relative frequency (% with confidence interval) of surgical procedures for IAAA repair according to type and degree of urgency, from 2008 to 2017.

	**Open**		**Endovascular**			***p*** ^*^
	**Urgent**	**Elective**	**Urgent**	**Elective**	**Total**	
2008	48 (16% - 11.8; 20.1)	76 (25.25% - 20.3; 30.2)	44 (14.6% - 10.6; 18.6)	133 (44.2% - 38.6; 49.8)	301	0.076
2009	44 (15% - 10.9; 19.1)	53 (18.1% - 13.7; 22.5)	60 (20.5% - 16.9; 25.1)	136 (46.4% - 40.7; 52.1)	293	
2010	49 (16.4% - 12.2; 20.6)	60 (20.1% - 15.5; 24.6)	66 (22.1% - 17.4; 26.8)	124 (41.5% - 35.9; 47.1)	299	
2011	46 (14.3% - 10.5; 18.2)	46 (14.3% - 10.5; 18.2)	93 (29% - 24; 33.9)	136 (42.4% - 37; 47.8)	321	
2012	49 (14.3% - 10.6; 18)	64 (18.7% - 14.6; 22.8)	95 (27.8% - 23; 32.5)	134 (39.2% - 34; 44.4)	342	
2013	32 (10.8% - 7.2; 14.3)	54 (18.2% - 13.8; 22.6)	67 (22.6% - 17.8; 27.3)	144 (48.5% - 42.8; 54.2)	297	
2014	23 (9.31% - 5.7; 12.9)	56 (22.7% - 17.4; 27.9)	47 (19% - 14.1; 23.9)	121 (49% - 42.8; 55.2)	247	
2015	30 (14.6% - 9.7; 19.4)	40 (19.4% - 14; 24.8)	40 (19.4% - 14; 24.8)	96 (46.6% - 39.8; 53.4)	206	
2016	16 (9.36% - 5; 13.7)	33 (19.3% - 13.4; 25.2)	33 (19.3% - 13.4; 25.2)	89 (52% - 44.6; 59.5)	171	
2017	46 (21.3% - 15.8; 26.8)	31 (14.3% - 9.7; 19)	41 (19% - 13.8; 24.2)	98 (45.4% - 38.7; 52)	216	
**Total**	**383 (14.2% - 12.9; 15.5)**	**513 (19% - 17.6; 20.5)**	**586 (21.8% - 20.2; 23.3)**	**1211 (45% - 43.1; 46.8)**	**2693**	

* Chi-square test for trend.

We observed a higher number of endovascular surgeries in all years evaluated. In total, we found that 66.73% of the surgeries were endovascular (of which 21.76% were emergency cases and 44.97% were elective cases), and 33.27% of all surgeries were performed by open techniques (14.22% urgent cases and 19.05% elective cases). The number of elective surgeries was higher than the number of emergency surgeries (64.02% of all surgeries were elective admissions). There was no tendency to change techniques over the years (p = 0.076).

Table 2 presents the numbers of procedures and mortality rates per hospital unit.

**Table 2:** Absolute and relative frequencies of procedures and mortality (% with confidence interval), by hospital unit.

Hospital	Total procedures	Total mortality, n (%)	Open group mortality		Endovascular group mortality	
			Elective	Urgent	Elective	Urgent
			mortality/ procedures (%)	mortality/ procedures (%)	mortality/ procedures (%)	mortality/ procedures (%)
1	635	21 (3.3% - 3.3; 3.32)	8/179 (4.47% - 4.42; 4.52)	4/30 (13.33% - 12.58; 14.09)	5/404 (1.24% - 1.23; 1.24)	4/22 (18.18% - 16.86; 19.51)
2	536	77 (14.37% - 14.32; 14.4)	23/169 (13.61% - 13.47; 13.75)	37/127 (29.13% - 28.82; 29.45)	2/139 (1.44% - 1.42; 1.46)	15/101 (14.85% - 14.61; 15.1)
3	459	60 (13.04% - 13.02; 13.12)	4/15 (26.67% - 24.12; 29.21)	36/122 (29.51% - 29.18; 29.84)	2/60 (3.33% - 3.23; 3.44)	18/262 (6.87% - 6.82; 6.92)
4	428	19 (4.44% - 4.42; 4.46)	11/77 (14.29% - 13.98; 14.6)	0/1 (0)	8/347 (2.31% - 2.29; 2.32)	0/3 (0)
5	298	59 (19.8% - 19.69; 19.9)	26/57 (45.61% - 44.77; 46.46)	0/0 (0)	31/220 (14.09% - 13.98; 14.2)	2/21 (9.52% - 8.72; 10.32)
6	227	43 (18.94% - 18.81; 19.07)	2/13 (15.38% - 13.43; 17.34)	26/81 (32.1% - 31.57; 32.62)	2/17 (11.76% - 10.57; 12.96)	13/116 (11.21% - 11.04; 11.37)
7	99	8 (8.08% - 7.93; 8.23)	0/3 (0)	3/21 (14.29% - 13.15; 15.42)	0/22 (0)	5/53 (9.43% - 9.12; 9.75)
8	8	1	0/0	1/1	0/0	0/7
9	3	0	0/0	0/0	0/2	0/1
**Total**	**2693**	**288 (10.69% - 10.69; 10.7)**	**74/513 (14.42% - 14.38; 14.47)**	**107/383 (27.94% - 27.83; 28.04)**	**50/1211 (4.13% - 4.12; 4.14)**	**57/586 (9.73% - 9.7; 9.76)**
*p* ^*^			*0.019*			

* Mortality in the open group was statistically higher than in the endovascular group (p = 0.019);

Mann-Whitney test.

There were 288 in-hospital deaths (mortality of 10.69%). In-hospital mortality for endovascular procedures was lower than for open procedures, for both elective (4.13% versus 14.42%) and emergency admissions (9.73% versus 27.94%). The difference in mortality between the two techniques was statistically significant (p = 0.019). The hospital with the highest number of surgeries (n = 635) had the lowest in-hospital mortality (3.31%). The only hospital that had no in-hospital deaths performed very few surgeries (n = 3).

Table 3 presents the number of procedures classified by length of stay.

**Table 3:** Absolute and relative frequency of procedures according to the number of days (time) in hospital.

	**Length of stay**	**< 1 day**	**1 day**	**2 days**	**3-7 days**	**>7 days**	***p*** ^*^
Open group	Elective	9 (1.8)	12 (2.3)	10 (1.9)	61 (11.9)	421 (82.1)	<0.001
	Urgent	22 (5.7)	32 (8.4)	16 (4.2)	82 (21.4)	231 (60.3)	
Endo group	Elective	18 (1.5)	30 (2.5)	177 (14.6)	519 (42.9)	467 (38.6)	
	Urgent	11 (1.9)	25 (4.3)	32 (5.5)	299 (51)	219 (37.4)	
	Total	60 (2.4)	99 (3.9)	235 (9.4)	755 (30.3)	1338 (53.7)	

* The number of days in hospital for open surgery was statistically higher than for endovascular surgery (p <0.001);

Mann-Whitney test.

We observed that most procedures had a length of stay exceeding 7 days (n = 1338). However, if we analyzed the techniques separately, we found that most endovascular procedures had a length of stay of 3 to 7 days, while a majority of the open procedures had a length of stay of more than 7 days. The number of days of hospitalization for open surgery was significantly higher than for endovascular surgery (p <0.001).

**Figure 1:**
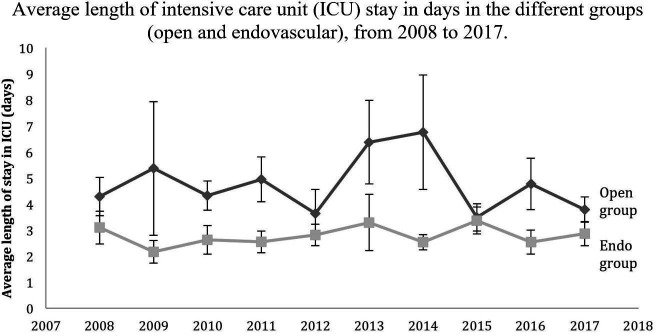
Average length of intensive care unit (ICU) stay in days in the different groups (open and endovascular), from 2008 to 2017.

Figure 1 illustrates the average length of ICU stay (in days) by patient group (open and endovascular).

The only statistically significant difference found was that patients undergoing endovascular treatment had a shorter mean ICU stay than patients undergoing open procedures (p <0.001).

Table 4 presents the sums in dollars paid by the SUS per hospital establishment.

**Table 4:** Values paid by the SUS, in US dollars, per hospital establishment.

Hospital	Total amount	Open amounts		Endovascular amounts		Amount per open treatment		Amount per endovascular treatment	
		Elective	Urgent	Elective	Urgent	Elective	Urgent	Elective	Urgent
1	5,747,417.82	314,275.36	54,502.60	5,086,920.90	291,718.96	1,755.73	1,816.75	12,591.39	13,259.95
2	3,919,407.00	383,169.37	453,906.71	1,769,056.37	1,313,274.55	2,267.27	3,574.07	12,727.02	13,002.72
3	4,695,813.56	27,724.50	410,956.70	738,935.71	3,505,146.58	1,848.30	3,368.50	12,315.59	13,378.42
4	4,564,416.69	282,769.04	2,846.36	4,245,058.28	33,743.01	3,672.32	2,846.36	12,233.60	11,247.67
5	2,732,240.99	133,265.40	0.00	2,368,941.44	230,034.15	2,337.99	0.00	10,767.92	10,954.00
6	2,058,464.35	38,193.09	323,524.10	187,589.12	1,509,158.04	2,937.93	3,994.12	11,034.65	13,009.99
7	1,000,411.92	10,240.65	63,395.88	298,313.60	628,461.78	3,413.55	3,018.85	13,559.71	11,857.77
8	76,246.59	0.00	928.36	0.00	75,318.23	0.00	928.36	0.00	10,759.75
9	41,185.93	0.00	0.00	27,883.16	13,302.77	0.00	0.00	13,941.58	13,302.77
**Total**	**24,835,604.84**	**1,189,637.41**	**1,310,060.71**	**14,722,698.59**	**7,600,158.07**	**2,318.63**	**3,420.10**	**12,157.35**	**12,969.12**

The total amount of money paid to the hospitals by the SUS was $24,835,604.84 for the total number of procedures. The average sums paid per procedure were as follows: $ 2,318.63 per elective open surgery; $ 3,420.10 for emergency open surgery; $ 12,157.35 for elective endovascular surgery, and $ 12,969.12 for urgent endovascular surgery. The comparison between elective and urgent open surgery found no difference in cost (p = 0.666). The same occurred for endovascular surgery (p = 0.796). However, the cost of open surgery was statistically lower than the cost of endovascular surgery, regardless of whether urgent or elective (p <0.001).

## DISCUSSION

The endovascular technique was used more frequently, especially in elective surgeries, requiring larger financial transfers from the SUS. One limitation of our study is that the database used only enables analysis of hospitals accredited by the public healthcare system to perform the procedures studied. Considering elective surgeries in particular, it might be thought that these results are the best available on the public system, since accredited hospitals should, in theory, be the elite of the system. However, considering that some hospitals performed a small number of surgeries in the 10-year interval (or that mortality in elective surgeries was close to 40%), it can be argued that the accreditation process could be improved. The hospital with the lowest mortality (3.31%) was precisely the one that performed the greatest number of procedures (635). This suggests that high-volume centers, in fact, have a lower frequency of complications, which has been observed not only for IAAA,^14^ but also for other diseases and procedures.^15^

Regarding age, our findings are compatible with other studies that also observed a predominance of surgical treatments in elderly men.^16^^-^18 We noticed a decrease in the number of procedures over the years. The European literature has shown a decrease in the frequency of emergency aneurysm repairs, with an increase in elective repairs,^17^ in addition to a lower prevalence of aortic aneurysms over the decades.^19^ A similar decreasing trend has also been observed in the United States.^20^ The proportion of patients with a registered address outside of the city of São Paulo (36.3%) shows that there is considerable centralization of health resources in a single metropolitan city, attracting patients from other cities.

There was no trend to variation in techniques over the years, corroborating the fact that endovascular surgery for treatment of IAAA is already established and is the predominant method in the city of São Paulo. In a 2017 systematic review, similar data showed a predominance of elective endovascular treatments in different countries.^17^ We observed a higher proportion of elective surgeries (64.02%). However, in other countries, the prevalence of elective surgery is even higher (almost 80%).^21^

The finding of higher in-hospital mortality for open surgery is consistent with the literature showing higher perioperative mortality for open surgery compared to endovascular surgery.^4^^-^6 As the analysis is by hospital admissions, the mortality data we have access to is in-hospital data only. Thus, we were unable to contribute information to the debate regarding the long-term superiority of these two techniques (or to the EVAR-1 study, which demonstrated lower survival in long-term endovascular-operated patients, especially the late rupture of some patients with stent graft, nor can we align with the DREAM or OVER studies, which, after many years, do not show better results for either technique).^4^^-^6

The morbidity of endovascular repair is less intense than open surgery, and even nephrotoxic iodinated contrast (which could be a disadvantage of the endovascular technique) can be replaced by alternative contrasts such as carbon dioxide.^22^

Although open surgery has been well-established and has achieved good results for decades in treating IAAA, with the growth of endovascular treatment, more cases with more complex and unfavorable anatomy are reserved for open treatment,^23^ especially when our public system does not have easy access to fenestrated or branched prostheses, because they are substantially more expensive than “conventional” stent grafts. We believe that this is why our data differ from the 2018 systematic review, which did not observe an increase in mortality in patients treated with the open technique after the beginning of the endovascular era.^24^

It was not unexpected that overall mortality would be higher in urgent hospital admissions (compared to elective). Nonetheless, the comparison of mortality in urgent surgeries between open and endovascular techniques merits discussion: if, on the one hand the endovascular technique is less morbid, on the other hand the lower mortality associated with its use may also be related to the lower severity of the patients (i.e., an aneurysm with pain that is hemodynamically stable is an emergency hospitalization, but the patient is able to wait longer for stent grafts to become available). Although there is one hospital in the city that is equipped to treat hypotensive (and therefore more severe) patients,^25^ at other hospitals that do not have the same infrastructure, patients arriving *in extremis* are more likely to undergo open procedures, which will impact mortality.

Length of stay can be used as a measure of healthcare efficiency.^26^^,^^27^ We found a difference in the length of stay between the two groups. Stays were shorter for patients who underwent endovascular procedures, with a length of stay of 3 to 7 days in most cases. This advantage of endovascular repair in terms of length of stay has already been proven in the literature. A retrospective analysis of the National Inpatient Sample (NIS) from 2000 to 2010 comparing EVAR and open repair reported a significantly shorter median length of stay after endovascular treatment.^28^

In addition to a shorter length of stay, the literature also shows shorter ICU stays for endovascular procedures.^4^ We observed the same in our sample. A 2019 retrospective analysis using the American College of Surgeons database found that emergency surgery was associated with longer ICU stays, as well as other factors such as aneurysm rupture or postoperative pneumonia.^29^ However, we did not observe a statistically significant difference in the length of ICU stay between elective and urgent admissions. One hypothesis is that patients who died early (i.e., in the first days of the ICU stay) lowered the average length of stay, making the averages comparable between the group of elective patients and those who were admitted on an urgent basis.

The SUS paid higher monetary values for endovascular surgery than for open surgery. A 2016 systematic review confirmed the higher cost of endovascular surgery and EVAR was found to be more cost-effective in specific patient groups, such as those at high surgical risk.^30^ In the Brazilian context, the literature also shows that EVAR is more expensive.^31^^,^^32^ One of the limitations in our analysis is the fact that the SUS has a fixed billing table for procedures that often does not reflect the amounts actually spent on the procedures.

## CONCLUSION

This study offers an initial description of in-hospital results and the profile of IAAA treatment in the city of São Paulo. The cost of treating IAAA is probably higher with EVAR than with open surgery, notwithstanding the limitations concerning an incomplete evaluation of financial costs. Endovascular surgery was much more frequent than open surgery.

## Supplementary Material

Supplementary material accompanies this paper.

This material is available as part of the online article from https://www.scielo.br/j/jvb

## Appendix A Algorithm.

Description of the algorithm developed in the applications:

1. Insertion of the procedures of interest into a table of search configuration;

2. Definition of the item to be displayed in the rows and columns of the table sought;

3. Definition of the data content of each case (total value of AIH’s paid, Total Value, Days in ICU and others);

4. Selection of the period of interest;

5. Selection of fields of interest in the session “SELECTIONS AVAILABLE” of Tabnet.

6. Enter the procedure in the “Performed procedure” field;

7. Request for data;

8. Wait until the page is fully loaded;

9. Request to download the table generated in a file with the Comma- separated values (CSV);

10. Wait until the file is fully downloaded;

11. Rename the file to the name of the procedure;

12. Removal of note and caption information;

13. Rearranging tables for use in Excel or other software; 14. Return to the initial selection form.
